# Primary Invasive Squamous Cell Carcinoma of the Vagina Presenting Three Decades After Total Hysterectomy for Benign Endometriosis: A Case Report

**DOI:** 10.7759/cureus.95242

**Published:** 2025-10-23

**Authors:** Tara McKenna, Kylie Pfeifer, Ariana Tiberi, Wanda I Torres

**Affiliations:** 1 Dr. Kiran C. Patel College of Osteopathic Medicine, Nova Southeastern University, Fort Lauderdale, USA; 2 Obstetrics and Gynecology, Suncoast Women's Care, Trinity, USA; 3 Dr. Kiran C. Patel College of Osteopathic Medicine, Nova Southeastern University, Clearwater, USA

**Keywords:** hpv-16, hysterectomy, primary vaginal cancer, squamous cell carcinoma, vaginal intraepithelial neoplasia

## Abstract

A 57-year-old woman (G2P2) with a history of total hysterectomy for endometriosis 30 years earlier, presented for a routine gynecological examination without complaints. Speculum examination revealed an erythematous, raised plaque at the right lateral fornix, 2-3 cm from the vaginal cuff. Pathology from the lesion demonstrated high-grade squamous intraepithelial lesion (HSIL) with HPV-16 positivity. Colposcopic biopsies from the 2, 7, 9, and 11 o’clock positions confirmed HSIL, with half of the samples suspicious for invasion. The patient underwent a partial vaginectomy, and histopathology revealed a moderately differentiated, HPV-associated invasive squamous cell carcinoma (SCC) with lymphovascular invasion. Margins were negative. This case demonstrates a unique case of primary vaginal cancer decades after hysterectomy for benign disease, underscoring the importance of continued gynecological surveillance in hysterectomy patients with previously benign findings.

## Introduction

Primary vaginal cancer is one of the rarest gynecological cancers and most commonly results from metastasis of adjacent gynecologic structures. Less commonly, metastasis from distant sites, such as the breast or pancreas, can also occur. Primary vaginal cancer originates in the vaginal tissue itself, whereas secondary (or metastatic) vaginal cancer arises from the spread of malignancy from other organs. Incidence and risk increase with age, with more than 50% of all patients diagnosed in the seventh decade of life [1-3]. High-risk human papillomavirus (HPV) types 16 and 18 are oncogenic and are the most significant risk factor for malignant transformation into squamous cell carcinoma [1,2,4-6]. 

Incidence of primary vaginal cancer after hysterectomy has been reported in patients who have both benign and malignant uterine pathologies. However, little research exists on primary vaginal cancer occurrence in cases of benign uterine conditions [7], with only one known literature review documenting eight studies with a total of 56 cases [8]. This report describes the case of an asymptomatic patient with a previously benign gynecological history and a diagnosis of primary malignant neoplasia of the vagina.

## Case presentation

An otherwise healthy 57-year-old woman, gravida 2 para 0002 (G2P2), presented for gynecological maintenance examination. Gynecological medical history was significant for total abdominal hysterectomy with bilateral salpingo-oophorectomy 30 years ago for definitive treatment of endometriosis. She denied any abnormal or post-coital vaginal bleeding. There was no history of abnormal Pap smears; her last screening seven years prior to presentation was normal. Additional history included a BMI of 26.1 kg/m^2^, a former 20-pack-year smoking history, moderate alcohol use, genital herpes simplex, two vaginal births, and post-menopausal hormone replacement therapy. She was adherent to screening examinations, including yearly mammograms, screening colonoscopies, and Pap smears every five years, all of which were negative.

Female genitalia exam revealed vulvovaginal changes consistent with postmenopausal vaginal atrophy. The speculum examination demonstrated a raised erythematous plaque on the lateral aspect of the vaginal cuff, and Pap smear sampling was obtained. Workup revealed high-grade squamous intraepithelial lesions (HSIL), HPV 16+ pathology of the plaque, and subsequent colposcopy was performed, which revealed HSIL at the 2, 7, 9, and 11 o’clock positions of the vaginal cuff. The lesions had negative uptake with Lugol’s iodine. The remaining vaginal exam was unremarkable.

**Figure 1:**
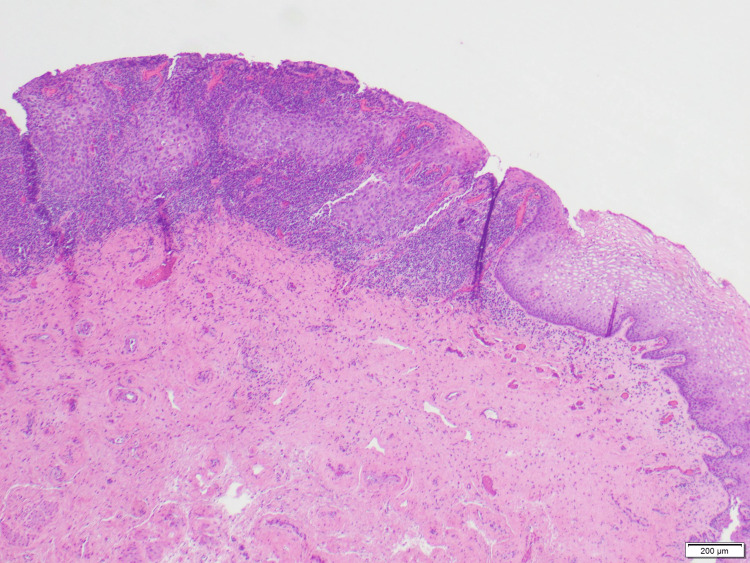
Squamous cell carcinoma in situ (HSIL, CIN3) with extension into the endocervical glands is shown alongside normal squamous epithelium. HSIL: high-grade squamous intraepithelial lesion; CIN3: cervical intraepithelial neoplasia grade 3

**Figure 2:**
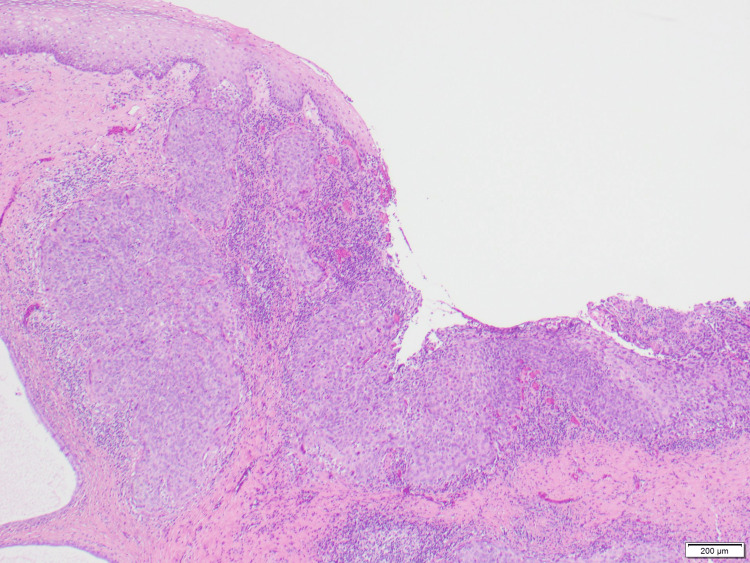
Normal squamous epithelium alongside in situ and invasive squamous cell carcinoma with growth into the endocervical glands and stroma.

**Figure 3:**
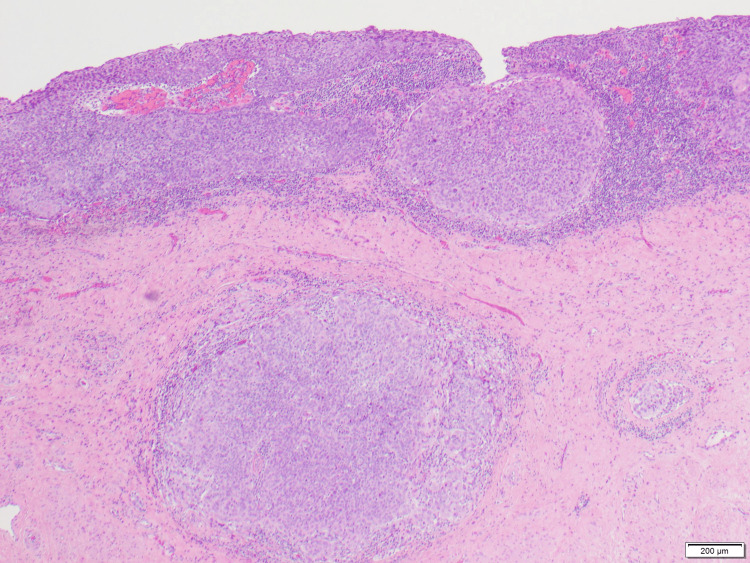
In situ and invasive squamous cell carcinoma.

**Figure 4:**
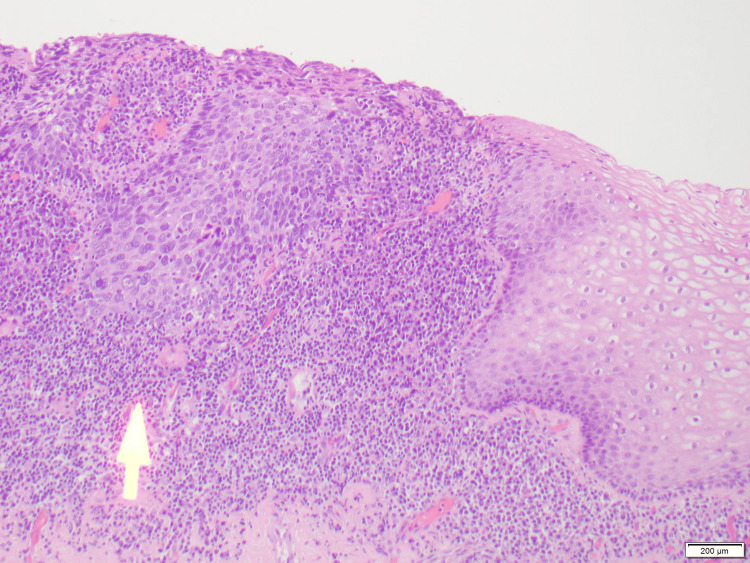
Increased power. In situ squamous cell carcinoma.

**Figure 5:**
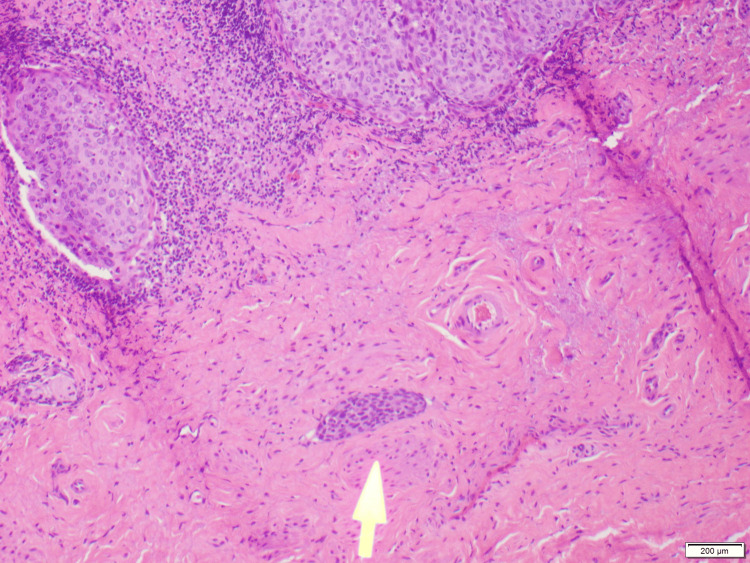
Invasive squamous cell carcinoma with lymphovascular invasion. Yellow arrow shows focus of tumor within a lymphatic space.

**Figure 6:**
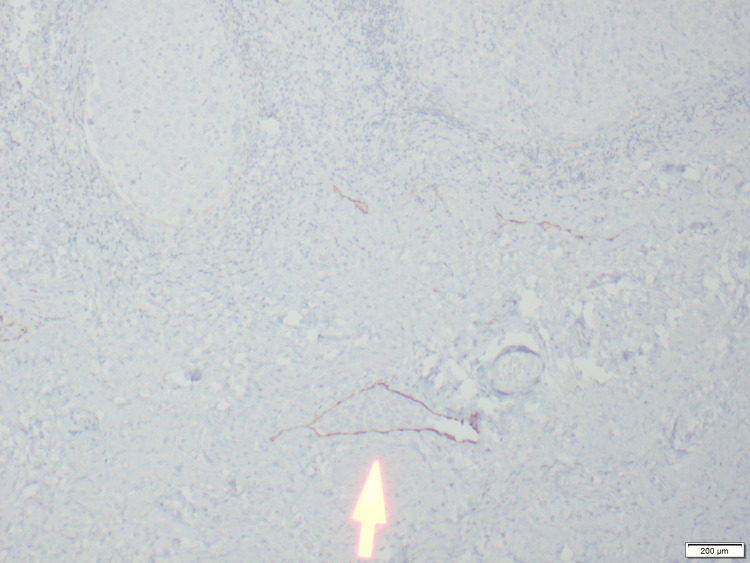
D2-40 immunostain of Figure 5 highlighting lymphatic endothelial cells lining the vessel (low power), marking lymphatic endothelium to confirm lymphovascular invasion by the tumor.

**Figure 7:**
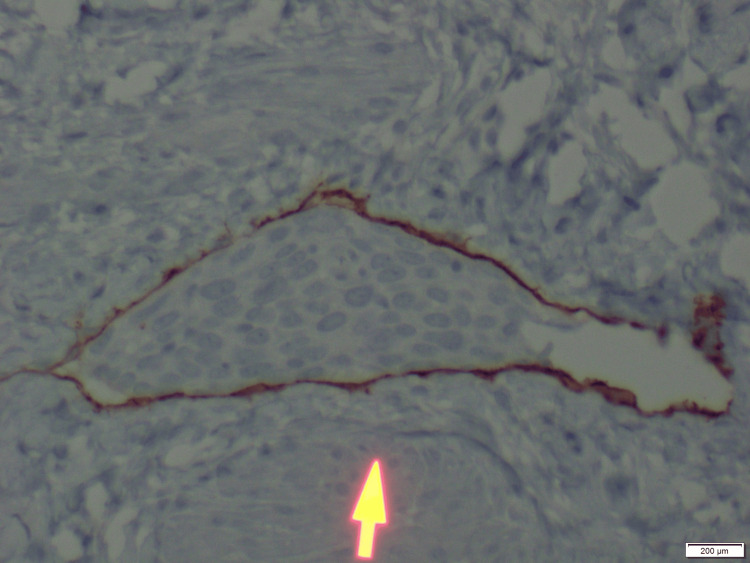
D2-40 immunostain of Figure 5 highlighting lymphatic endothelial cells lining the vessel (high power), marking lymphatic endothelium to confirm lymphovascular invasion by the tumor.

The patient underwent colposcopy and biopsy, with pathology confirming HSIL. A month later, on the recommendation of her Gynecologic Oncologist, she underwent partial vaginectomy, which revealed a 7 x 3 mm moderately differentiated, HPV-associated invasive squamous cell carcinoma with lymphovascular invasion (Figures 1-7). She was diagnosed with a malignant neoplasm of the vagina (Stage IA FIGO (Fédération Internationale de Gynécologie et d'Obstétrique)) grade 2 squamous cell carcinoma, and surgical margins were negative. Three months after this, she continues to follow up with Gynecologic Oncology, including PET-CT imaging, for ongoing management. 

## Discussion

Primary vaginal cancer is a rare subtype of gynecologic malignancy, representing only 1-2% of cancer in the female reproductive tract [1,2]. There are many categories of cancer, differentiated by the predominant cell or tissue where the cancer originates, and include: squamous cell, adenocarcinoma, melanoma, sarcoma, and lymphoma. The former, squamous cell carcinoma (SCC), is the most common form of vaginal cancer, accounting for 90% of cases [1]. Most cases of primary vaginal cancer appear in the seventh and eighth decade of life, and only a small percentage of women are diagnosed under the age of 50. HPV 16-induced neoplasia is a known risk factor for the development of primary vaginal cancer due to inhibition of cell cycle modulator proteins E6 and E7 [1,4-6]. Additionally, in utero exposure of diethylstilbestrol (DES), a synthetic estrogen historically used to prevent miscarriages and other pregnancy complications until 1971, has been associated with increased incidence of clear cell adenocarcinoma in those exposed to the drug [1,9]. 

Symptoms of primary vaginal cancer vary. Patients may notice abnormal changes in vaginal discharge such as odor, or new onset of vaginal bleeding. However, as many as 20% of patients are asymptomatic and therefore require a thorough physical examination for discovery [1,5,9]. Although no routine screening indications for vaginal cancer exist, cervical cytology and evidence of cervical lesions are indicators for further evaluation of vaginal tissue [7,10-12]. For women who have had a hysterectomy for benign disease (e.g., endometriosis), long-term vaginal surveillance is suggested for the unlikely, but potentially dangerous, development of vaginal neoplasia [7,10-12]. 

Treatment of vaginal cancer is influenced by the stage of disease and location, with modalities including local excision, surgery, radiation, chemotherapy, and lymphadenectomy [13-15]. Prognosis is dependent on various factors but can be most commonly predicted by stage, with stage I having the highest survival rates [12]. The presence of lymphovascular invasion is an additional adverse prognostic factor [4,16].

Primary vaginal cancer is extremely rare, particularly in the case of a prior benign hysterectomy [7,10]. As of today, we have identified only one systematic review from China [8] according to an English-language database search. Updated recommendations maintain that secondary prevention of vaginal cancer after hysterectomy for benign disease lacks sufficient evidence for routine screening [10-12], as hysterectomy for benign disease is not itself a recognized independent risk factor. 

Finally, it is necessary to consider the additional risk factors which predisposed the patient to the development of a gynecological malignancy. She endorsed a 20-year history of cigarette smoking, which may represent the strongest association to the development of disease [8]. 

## Conclusions

This is a case of primary vaginal cancer three decades after a benign hysterectomy for endometriosis. The patient’s unremarkable gynecologic history since surgery made the discovery of primary vaginal cancer unlikely. This case demonstrates the importance of continued gynecologic surveillance in patients with benign exams after a benign hysterectomy, despite the low occurrence of malignancy. It also highlights the role of HPV testing and physical examination in detecting clinically silent but high-grade lesions. Further research and larger studies are needed to clarify surveillance strategies for women with prior benign hysterectomy, particularly in the setting of HPV positivity. 
